# A Deep Learning Image Data Augmentation Method for Single Tumor Segmentation

**DOI:** 10.3389/fonc.2022.782988

**Published:** 2022-02-14

**Authors:** Chunling Zhang, Nan Bao, Hang Sun, Hong Li, Jing Li, Wei Qian, Shi Zhou

**Affiliations:** ^1^ College of Medicine and Biological Information Engineering, Northeastern University, Shenyang, China; ^2^ Department of Radiology, Affiliated Hospital of Guizhou Medical University, Guiyang, China; ^3^ College of Engineering, University of Texas at El Paso, El Paso, TX, United States

**Keywords:** data augmentation, tumor, segmentation, deep learning, breast cancer

## Abstract

**Purpose:**

Medical imaging examination is the primary method of diagnosis, treatment, and prevention of cancer. However, the amount of medical image data is often not enough to meet deep learning needs. This article aims to expand the small data set in tumor segmentation based on the deep learning method.

**Methods:**

This method includes three main parts: image cutting and mirroring augmentation, segmentation of augmented images, and boundary reconstruction. Firstly, the image is divided into four parts horizontally & vertically, and diagonally along the tumor’s approximate center. Then each part is mirrored to get a new image and hence a four times data set. Next, the deep learning network trains the augmented data and gets the corresponding segmentation model. Finally, the segmentation boundary of the original tumor is obtained by boundary compensation and reconstruction.

**Results:**

Combined with Mask-RCNN and U-Net, this study carried out experiments on a public breast ultrasound data set. The results show that the dice similarity coefficient (DSC) value obtained by horizontal and vertical cutting and mirroring augmentation and boundary reconstruction improved by 9.66% and 12.43% compared with no data augmentation. Moreover, the DSC obtained by diagonal cutting and mirroring augmentation and boundary reconstruction method improved by 9.46% and 13.74% compared with no data augmentation. Compared with data augmentation methods (cropping, rotating, and mirroring), this method’s DSC improved by 4.92% and 12.23% on Mask-RCNN and U-Net.

**Conclusion:**

Compared with the traditional methods, the proposed data augmentation method has better performance in single tumor segmentation.

## 1 Introduction

As we all know, cancer accounts for a large proportion of the diseases causing premature human death if noncommunicable conditions are not taken into account, and this trend will continue or increase in the future (1). Fortunately, the prevention and treatment of cancer through early detection can effectively reduce mortality (2, 3). In recent years, medical imaging has played an essential role in tumor screening, diagnosis, and treatment (4). In the past, the medical image was only regarded as a simple visual observation object (5). With the explosive development of deep learning in various application directions, medical imaging and medical image analysis have also been widely concerned (6). The weighted model through deep learning has been constructed to explore the deep features of the tumor image and further help for tumor target detection, classification, and prognosis prediction of unknown medical images, which can more effectively assist doctors in clinical diagnosis (7–10).

It has been proved that the data set’s size affects the performance of deep learning applied to the image analysis (11). With the increase in the amount of data, the deep learning model’s performance will be improved accordingly. However, unlike natural images, enough high-quality tumor medical images are not easy to obtain. It leads to the situation that the amount of data cannot meet the demand in applying deep learning of medical image analysis and even produce an overfitting effect. For this reason, some methods have been proposed to prevent overfitting, such as primary data augmentation, regularization, dropout, transfer learning, etc. (12). Nevertheless, effective data augmentation is the fundamental solution to the above problems (12).

There are many methods to augment image data. The most popular way is geometric transformation, including cutting, translation, rotation, mirror image, etc. The essence is to change pixels’ spatial position. Color space transformation changes the original spatial expression form of the image, which can be used for color casting and brightness adjustment (13), or the transformation and combination of different color spaces (14). The data augmentation method based on the disturbance is to add disturbance information to the original data to increase data, such as noise injection, blur, random erasure, and so on (15–17). In polyp segmentation, Sanchez-Peralta et al. (18) used the geometric transformation, color space transformation, and disturbance-based augmentation method. Compared with the non-augmentation way (on different data sets), it improved the intersection over union (IoU) by 6.58%, 13.24%, and 0.36%, respectively. The mixup data augmentation method combines two or more images. The composite image comprises all the input image pixels (linear mixing), such as mix up and pairing samples (19, 20), or part of the pixels (nonlinear mixing), such as the cut-mix proposed in the paper (21). Nishio et al. (22) applied a mixed data augmentation method to pancreas segmentation, improving the DSC by 8.6%. Based on learning data, the data augmentation uses a deep learning network to learn characteristics of the original data set and then expand it, such as a generative adversarial network (GAN) (23). Shi et al. (24) applied GAN to the pulmonary nodule segmentation, and its DSC value increased from 83.68% to 85.21%. However, these data augmentation methods have some limitations. For the GAN, it has the mode collapse problem, and GAN is also a deep learning network, which requires high-demand equipment support when expanding data (25). Traditional data augmentation methods have limited performance improvements for deep learning (26). For the data augmentation methods based on the color space transformation, the disturbance, and the mixup data, they have to change the pixel values of the original images, which will affect the segmentation tasks.

This paper proposed a novel data augmentation method, which used regional tumors to generate similar whole tumor images. When using a deep learning segmentation network, it can be applied to a relatively small image data set of single tumors. This method includes three parts. First, Horizontal & vertical cutting and mirroring augmentation (HVCMA) and diagonal cutting and mirroring augmentation (DCMA) along the tumor’s approximate center were conducted to obtain four times the number of original images. Then, the extended training set was sent into the deep learning network to acquire the segmentation model, and then the segmentation result of the extended testing set was obtained through the segmentation model. Finally, the boundary reconstruction was conducted on the four segmented images (originated from one tumor image) to get the original image’s corresponding tumor boundary.

Compared with the existing augmentation methods, the data samples augmented by the proposed method have the following characteristics: (1) Each sample comes from an actual image. (2) These samples have better independence than the traditional method. Unlike cropping, rotating, or mirroring, the adjacent pixel relationships in different samples are different. (3) The generated new tumor sample has good boundary information after two mirrorings from a quarter region of the tumor, conducive to accurate segmentation.

This paper’s sections were arranged as follows: Section 2 introduced the augmentation method and experimental arrangement. The experimental results were shown in Section 3 and discussed in Section 4. Finally, section 5 gave the conclusion of the work and summarized the future research.

## 2 Materials and Methods

### 2.1 Dataset

The data set used in this paper was provided by Al-Dhabyani et al. (27), including 780 breast cancer ultrasound images and their segmentation ground truths. In addition, we selected 503 single tumor images in the dataset to meet this paper’s research purpose, including 309 benign tumors and 194 malignant tumors.

In the experiment, we use 4-fold cross-validation to evaluate the performance of the deep learning segmentation model. That is, the whole data set was divided into four exclusive subsets, one subset (25%) was used as independent testing set each time, and the other three subsets were mixed as the training set (67.5%) and validation set (7.5%) of the deep learning network. **Table 1** shows the characteristics of the tumors in each fold.

**Table 1:** The characteristics of the tumors in each fold.

	Mean (pixels)	Interquartile range (pixels)	Median (pixels)	Number of benign cases	Number of malignant cases
Fold1	30154.00	38127.00	22863.00	73	52
Fold2	33902.00	40097.00	18371.00	73	52
Fold3	33287.00	41070.00	20719.00	73	52
Fold4	24889.00	27413.00	16364.00	83	45

### 2.2 Methods

**Figure 1:**
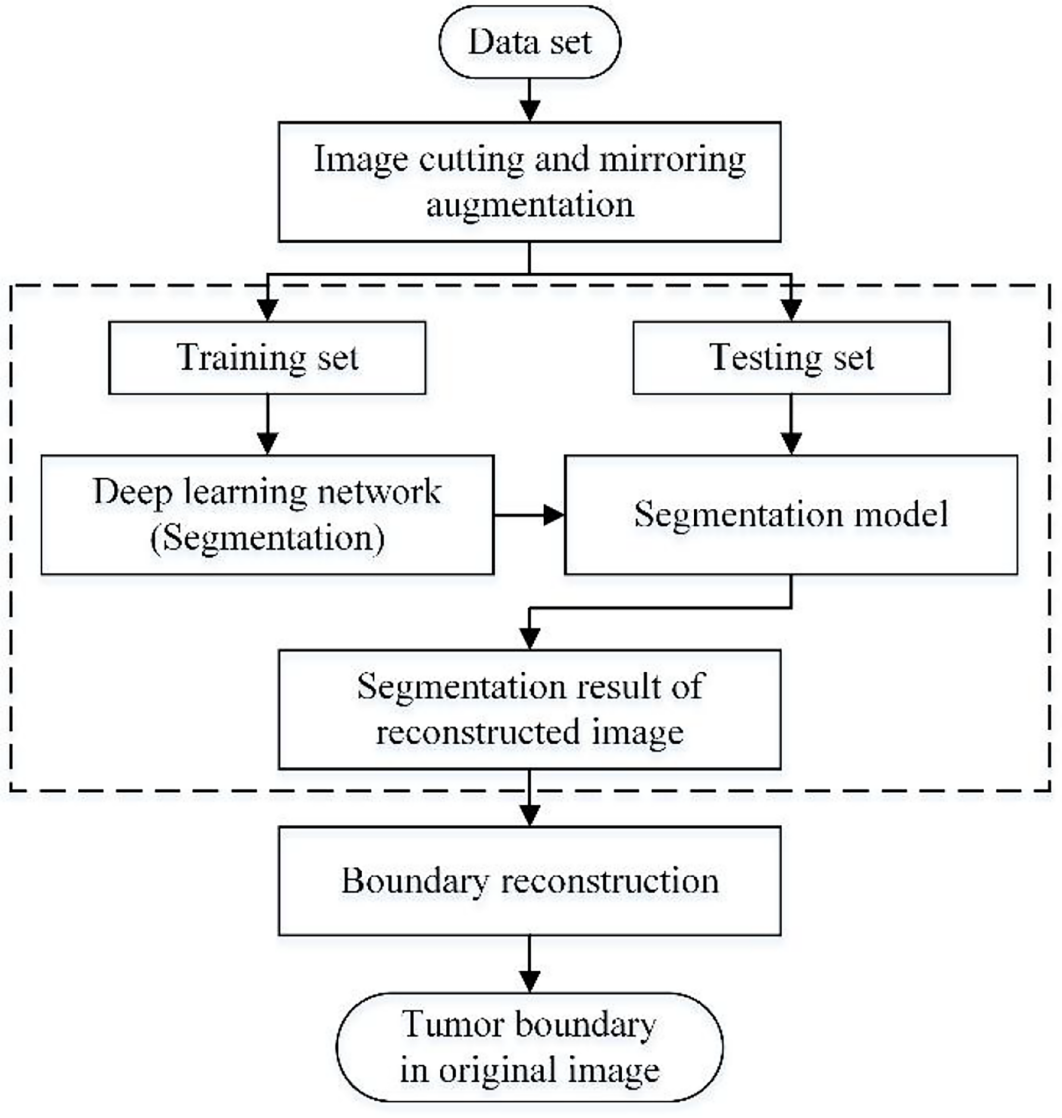
Flowchart of this method.

This paper’s data augmentation method is suitable for expanding the medical image data set with a single tumor, which combines cropping and mirroring in geometric transformation. Moreover, The traditional training set of deep learning applications is the union of the augmented data and the original data set (28). However, this method only takes the augmented data as the training set. Therefore, compared with the traditional way, it has one more step of boundary reconstruction. The flow chart of the method studied in this paper is shown in **Figure 1**, which mainly includes image cutting and mirroring augmentation, deep learning segmentation, and boundary reconstruction.

#### 2.2.1 Image Cutting and Mirroring Augmentation

The method of image augmentation differs in how to cut the image. This paper conducted two cutting methods: (1) dividing the image into four parts horizontally and vertically at the central point. (2) dividing the image into four parts diagonally at the central point. Next, the cut images are mirrored to obtain new images. The augmentation method using (1) is called HVCMA, and using (2) is called DCMA. We will introduce these two augmentation methods in detail.

##### 2.2.1.1 HVCMA

Let matrix *I* represents the original image, take a certain point as the center, and cut the image horizontally and vertically into four parts, represented by *A, B, C* and *D*. Then *I* is represented by *A, B, C* and *D*, as formula (1).


(1) $$I=\left[\begin{array}{ll} C & D\\ B & A \end{array}\right]$$

The double mirrored (horizontally and vertically) image of *A* is as follow:


(2) $$\tilde{A}=[\begin{array}{cc} A^{\prime \prime \prime } & A^{\prime \prime }\\ A^{\prime } & A \end{array}]$$

Where *A*′ is the horizontal mirror of *A*, *A*″ is the vertical mirror of *A*, *A*‴ is the diagonal mirror of *A*, and *Ã* is the generated image matrix composing a quarter of the original image and its three mirroring images.

**Figure 2:**
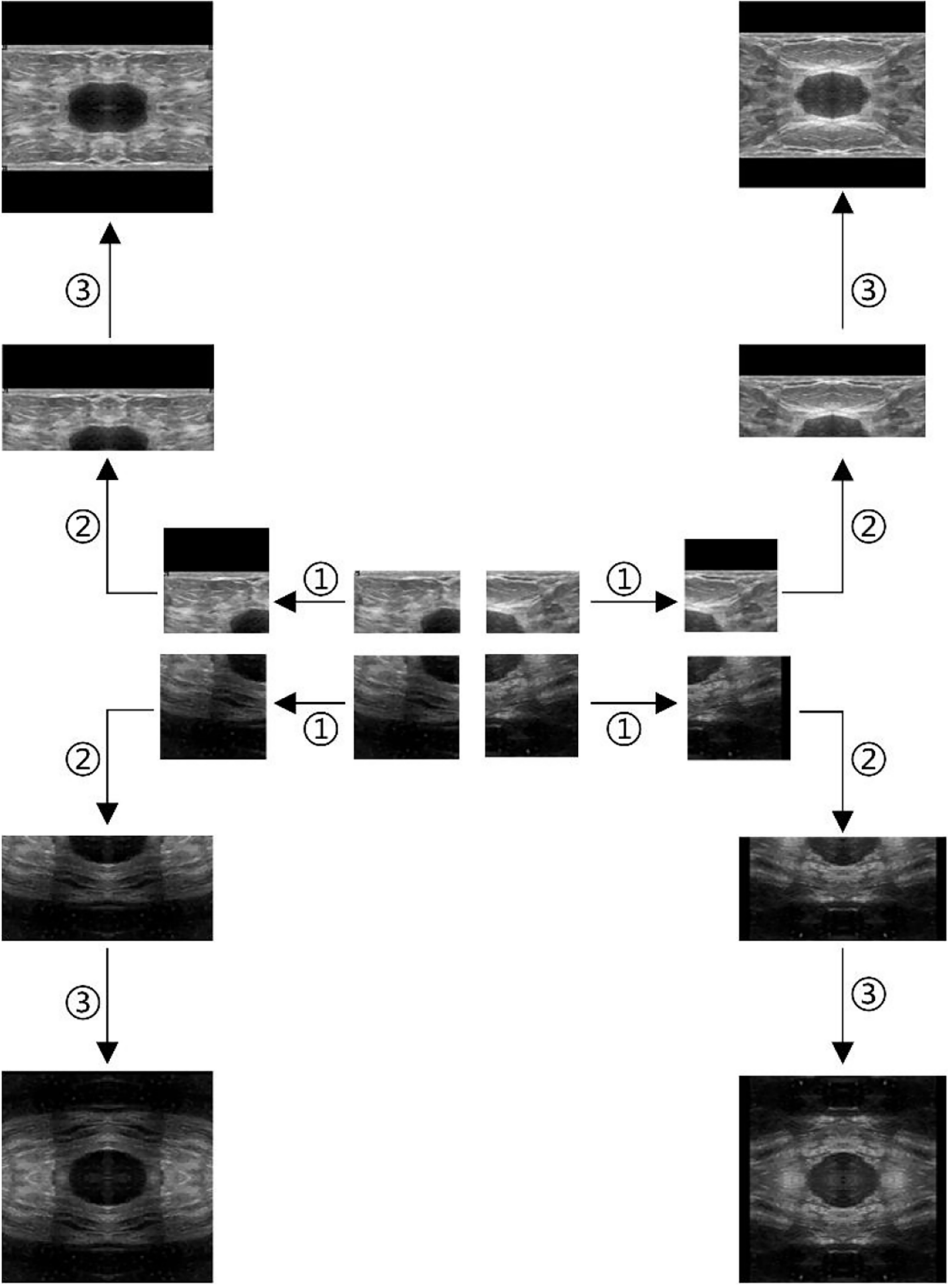
Augmentation by horizontal and vertical mirroring around the approximate tumor center. ① filling background of the cut image, ② horizontal mirroring, ③ vertical mirroring.

Take the ultrasound tumor image as an example. First, determine the cutting center point O (generally the approximate center of the tumor), cut the image vertically and horizontally along the center point, and get four images, as shown in **Figure 2**. However, the tumor is located at the edge in some cases. Moreover, cutting such an image will result in a large ratio of length to width of the generated images. To prevent this situation, we make the generated image a square, with its side length as the length of the longer side of the cutting part, and fill the missing pixel with 0, as shown in step ①. After getting the four cut images, mirror each one horizontally and vertically, and splice them into one image to get a set of four times expanded data, as shown in steps ② and ③.

##### 2.2.1.2 DCMA

When *I* is diagonally cut into four parts with point O, the image *I* can be expressed in equation (3).


(3) $$I=\left[C1\;\begin{array}{l} D1\\ B1 \end{array}\;A1\right]$$

Similarly, expand *A1* diagonally, we can get


(4) $$\bar{A}=\left[A1^{'''}\begin{array}{c} A1^{''}\\ A1^{'} \end{array}A1\right]$$

Where *A1'* and *A*″ are mirrors of *A1* along two diagonal lines, *A1‴* is the diagonal mirroring of *A1* and *Ā* is the generated image matrix composing a quarter of the original image and its three mirroring images.

**Figure 3:**
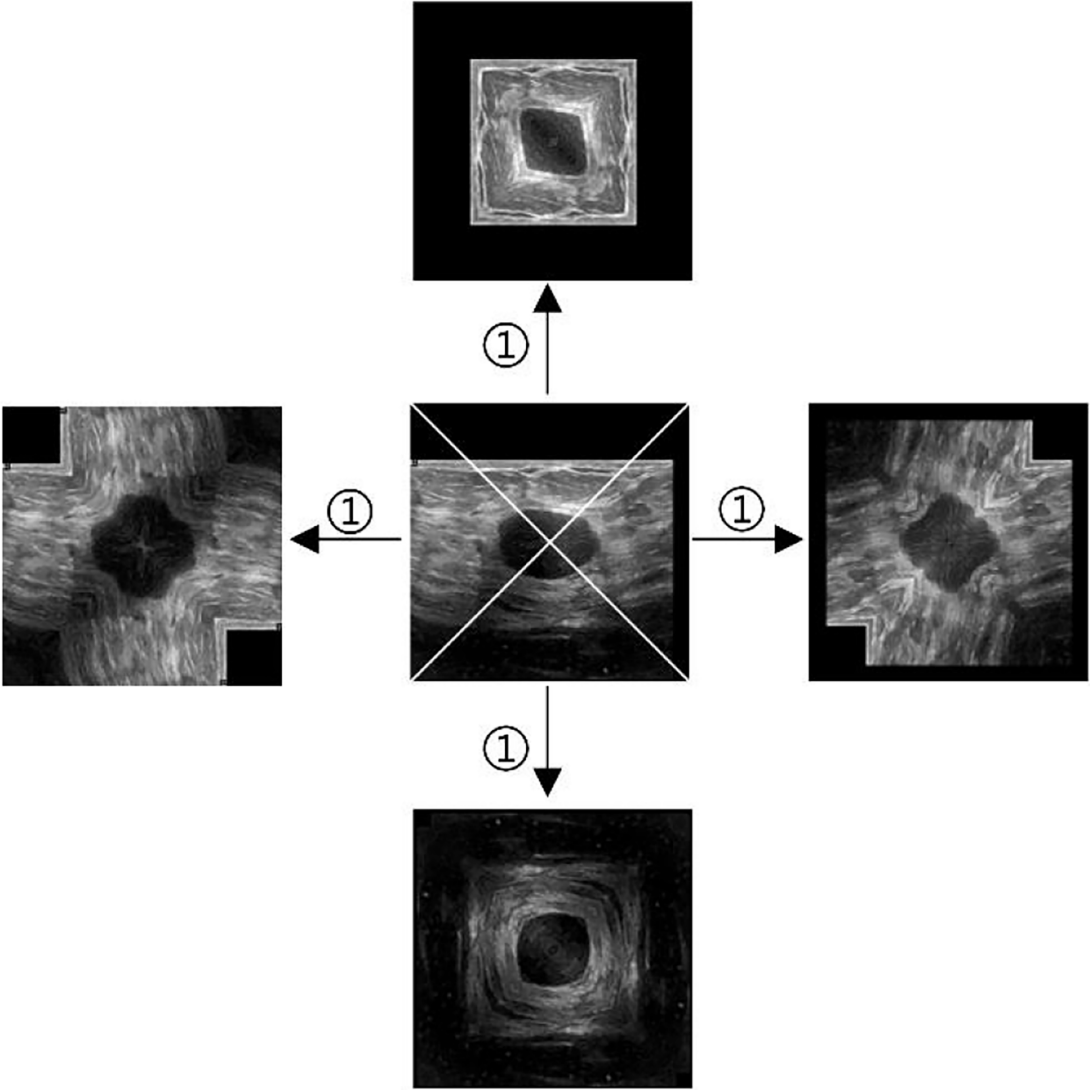
Diagonal augmentation. ① mirror and splice diagonally.

The same center point is used as the one in HVCMA. First, the image is supplemented as a square image centered on O, with the missing pixels filled with 0. Next, divide the tumor image into four parts diagonally, mirror each part of the image, and finally, get an additional set of four times expanded data. The way is shown in **Figure 3**.

#### 2.2.2 Deep Learning Segmentation

**Figure 4:**
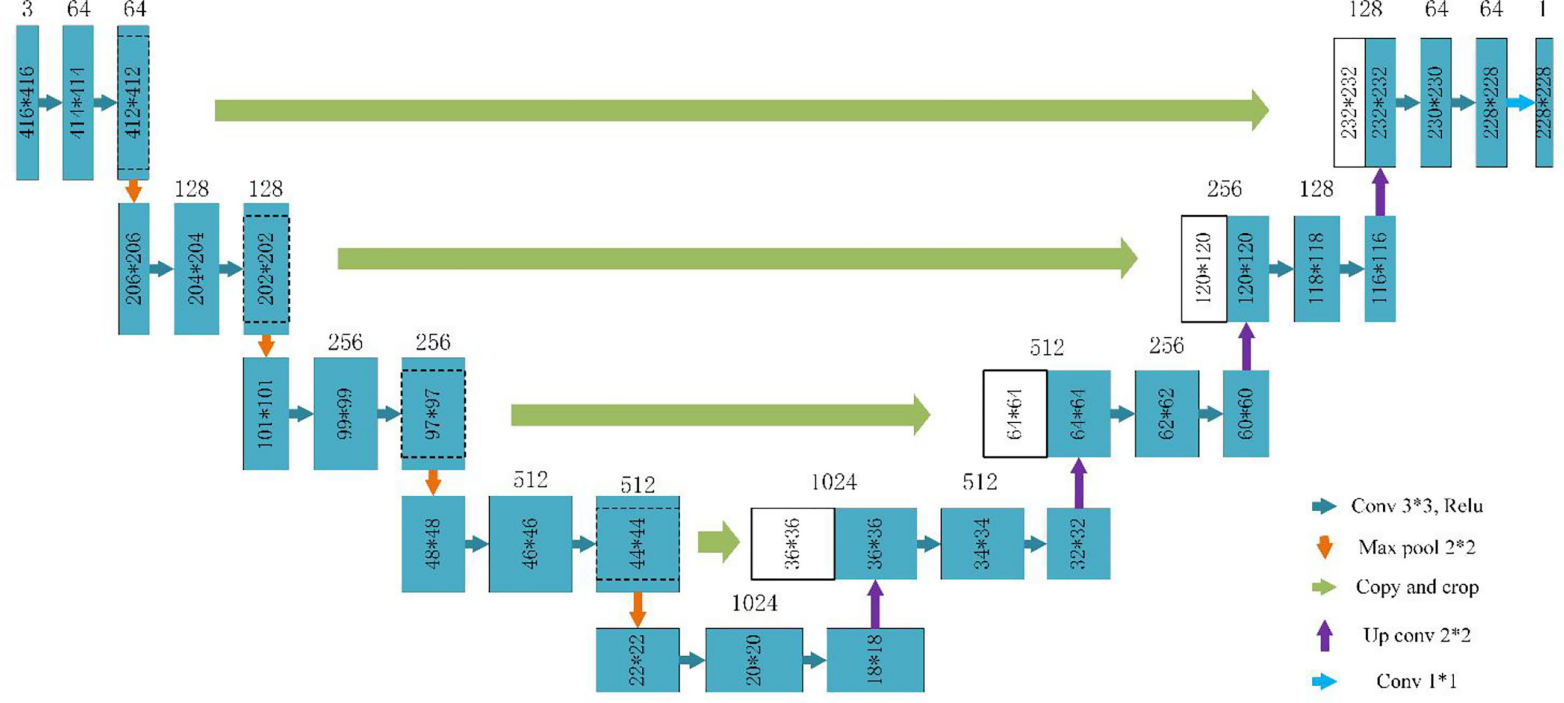
U-Net architecture based on (31).

**Figure 5:**
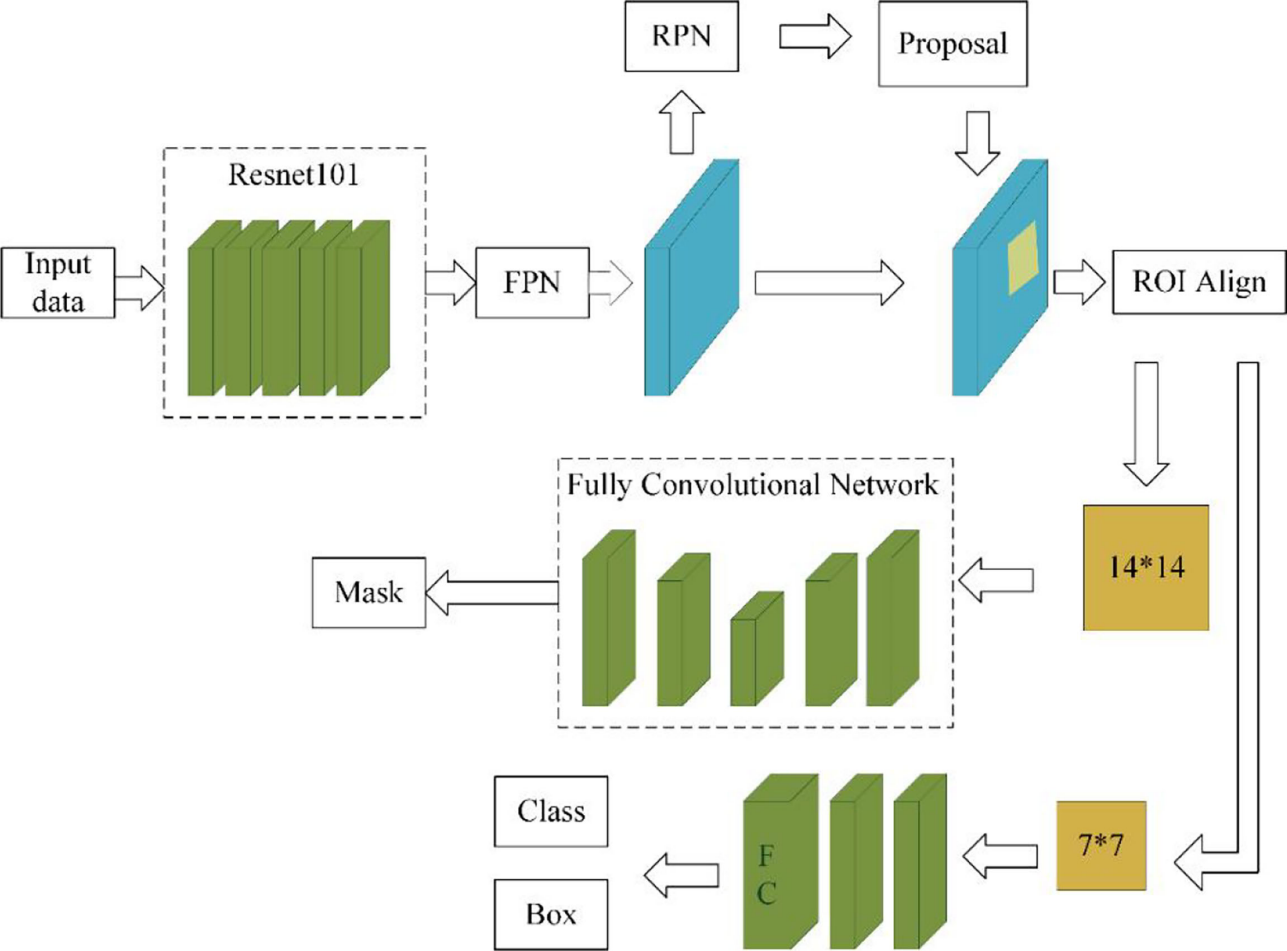
Mask-RCNN network architecture.

The data augmentation method studied in this paper can be applied in a deep learning segmentation network. There are many segmentation networks used in medical imaging analysis, such as FCN (29), SegNet (30), U-Net (31), FCDenseNets (32), ENet (33), Mask-RCNN (34), DeepLabv3+ (35) and so on. In our experiment, we chose two commonly used networks, Mask-RCNN and U-Net, which have excellent performance in medical image segmentation. The Mask-RCNN network is based on the region proposal network algorithm to obtain the feature map of the ROI before segmenting the target, while the U-Net is based on the encoding-decoding structure to segment the ROIs (36). The U-Net architecture is simple, and it is always considered as the baseline for the medical image segmentation tasks. **Figure 4** shows the network architecture of U-Net. Meanwhile, Mask-RCNN is also considered to be a simple, flexible, and general object instance segmentation framework (34). In our experiment, the Mask-RCNN program is provided by Abdulla (37) on GitHub. The network architecture is shown in **Figure 5**, ResNet 101 is used as the backbone, and the feature pyramid networks are used for the feature fusion. We initialize the model with the weights obtained by pre-training on the MS COCO data set (38).

The experiments of U-Net and Mask-RCNN both use Python 3.6 based on Keras and TensorFlow. The operating system is 64-bit Windows 10, the CPU is 3.70-GHz Intel(R) Core(TM) i7-8700K, and the GPU is NVIDIA GeForce GTX 1080Ti. The main parameters of the two networks in training are shown in **Table 2**.

**Table 2:** Parameters of the deep learning networks.

	Mask-RCNN	U-Net
Batch size	4	4
Learning rate	starting at 10^–3^, and decrease by one-tenth every thirty epochs in the non-augmented dataset and every ten epochs in the augmented dataset.	Starting at 10^–3^, and when the loss does not decrease after five epochs, the learning rate decreases by one-tenth.
Epoch	110 in the non-augmented dataset and 28 in the augmented dataset	110 in the non-augmented dataset and 28 in the augmented dataset
Input image format	RGB images (The size of the input images is not fixed)	RGB images (416*416*3)

**Figure 6:**
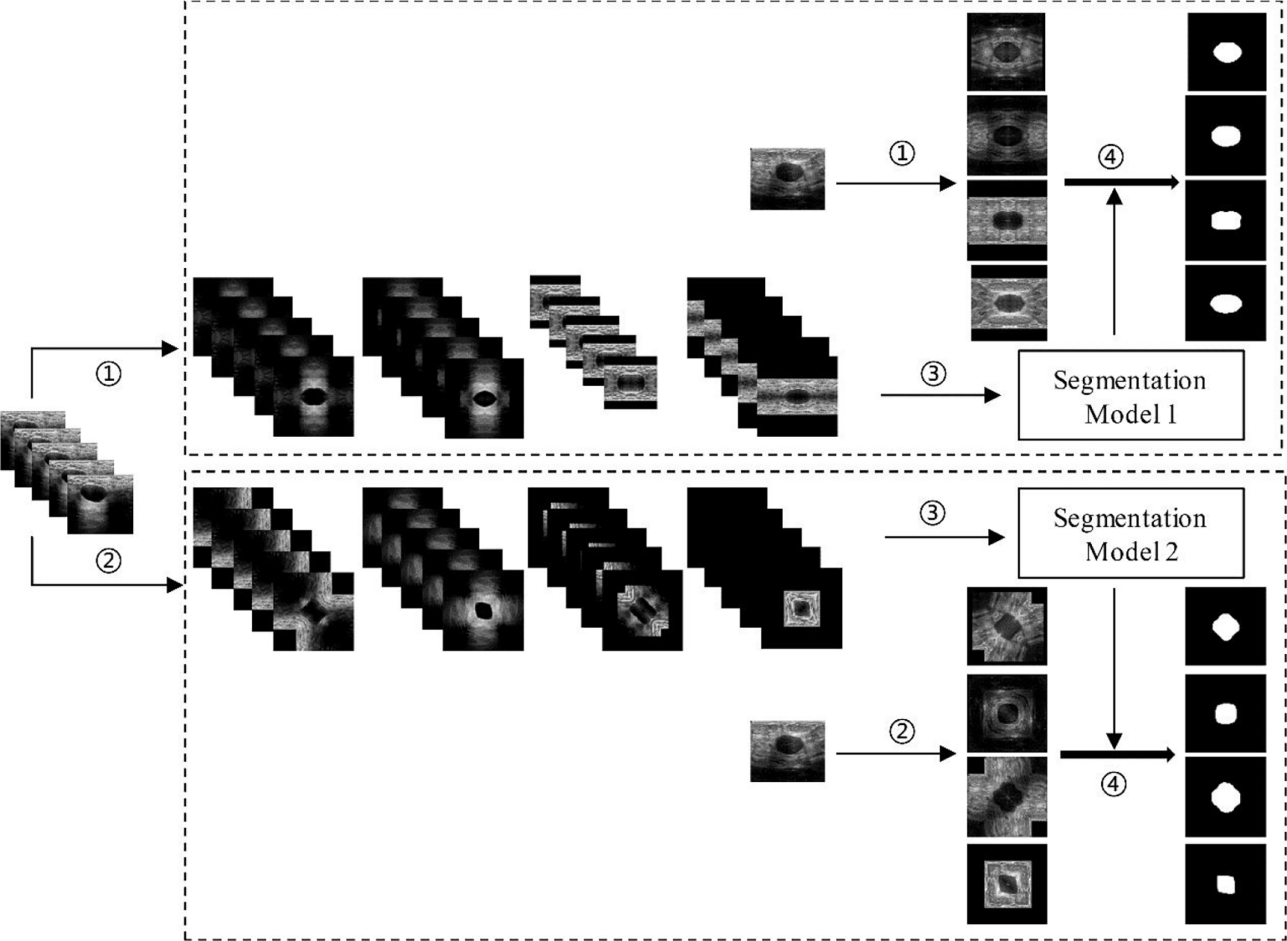
Deep learning segmentation based on different data augmentation methods. ① HVCMA, ② DCMA, ③ deep learning network training, ④ segmentation prediction of the testing case.

As mentioned above, the augmentation methods used in this paper were the HVCMA and the DCMA. Therefore, as shown in **Figure 6**, the original images (in testing set and training set) have been expanded by four times through step ① and step ②. Then, the augmented training data were sent to the deep learning segmentation network (step ③: Mask-RCNN or U-Net) to obtain segmentation model 1 and model 2 corresponding to step ① and ②. Finally, the segmentation results of the two augmented testing cases were obtained through their models in step ④.

#### 2.2.3 Boundary Reconstruction

Boundary reconstruction is to get the original tumor boundary from the results of the above deep learning segmentation model. The whole process is the reverse of data augmentation. Therefore, according to the data augmentation method, there are two boundary reconstruction processes, which we call single-mode boundary reconstruction. However, the images augmented by the HVCMA and DCMA methods may lose information at the cutting edge. To solve this problem, we combined the segmentation results of the above two segmentation models to carry out boundary reconstruction, which we call complex mode boundary reconstruction.

##### 2.2.3.1 Single-Mode Boundary Reconstruction

**Figure 7:**
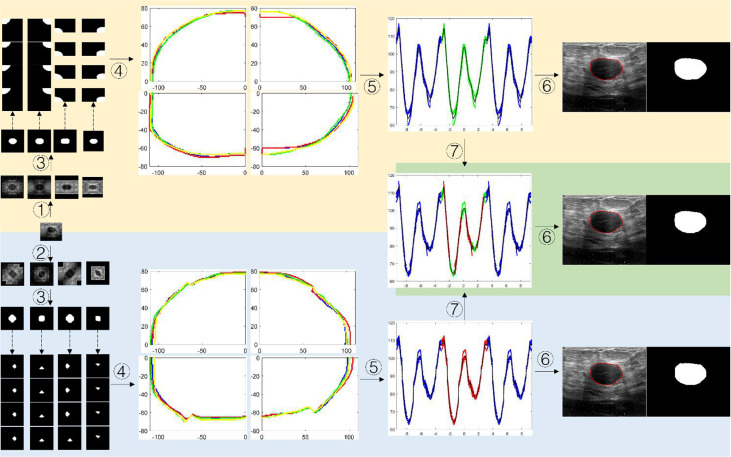
The schematic diagram of segmented boundary reconstruction for the testing image. ① HVCMA, ② DCMA, ③ deep learning segmentation prediction from model 1 or mode 2, ④ extracting the tumor boundaries and mapping them to the original image coordinates, ⑤ transforming the rectangular coordinates to the polar coordinates and expands the data, ⑥ transforming the boundary to the rectangular coordinate system after boundary fitting and interception, ⑦ compensating and expanding the boundary data).

**Figure 7**. shows the schematic diagram of the tumor boundary reconstruction process. For a testing case, the image was augmented four times by HVCMA or DCMA first. Then the segmentation results can be obtained using model 1 and model 2. See steps ①-③. Next, each binary image was inversely mirrored, refer to formulas (2) and (4), obtaining *A* from *A'*, *A″* and *A*‴, and *A1* from *A1*, *A1*″and *A1‴*, respectively, and four segmentation results are obtained for each quarter of the original tumor image, as shown in the dotted line step in **Figure 7**. Afterward, the tumor boundaries were extracted and mapped to the original image coordinate, as shown in step ④. The following steps were conducted since the tumor boundary should be a closed curve. We first transformed the tumor boundary coordinates to the polar coordinates to facilitate the processing. Generally, the data range in polar coordinates is [-π, π]. Since the tumor boundary at the endpoints may be divergent, the data points in the polar coordinates were expanded to triple period data, and the new data range is [-3π, 3π], as shown in step ⑤. After the curve fitting, the intermediate period’s fitting curve [-π, π] was extracted. It can be discretized and transformed into a rectangular coordinate system, and the tumor boundary can be obtained, as shown in step ⑥.

##### 2.2.3.2 Complex Mode Boundary Reconstruction

The segmentation results from model 1 and model 2 can be integrated to compensate for the information loss at the cutting position, which may form a new set of data, as shown in step ⑦ in **Figure 7**. We extracted the data points in range *U* in the diagonal augmentation and extracted other data points excluding *U* in the horizontal and vertical augmentation in [-π, π]. Then we combined two data groups into [-π, π] group, and the expression is shown as formula (6). Finally, step ⑥ was performed to obtain the compensated segmentation boundary and binary tumor image.

Let *g(x)* and *f(x)* represent the data relationship in polar coordinates of the segmented tumor boundary obtained by HVCMA and DCMA through deep learning segmentation models, and let *S* represents the interval [-π, π], then the data points can be expressed as:


(5) $$\{\begin{array}{l} g=g(x),x\in S\\ f=f(x),x\in S \end{array}$$

If the compensation data of reconstructed boundary is expressed by q the expression is as follow:


(6) $$q=\{\begin{array}{l} g(x),x\in U\\ f(x),x\in C_{S}U \end{array}$$

Where $U=\left\{\left[-\pi ,-\pi +h\right]\cup \left[-\frac{\pi }{2}-h,-\frac{\pi }{2}+h\right]\cup [-h,h]\cup \left[\frac{\pi }{2}-h,-\frac{\pi }{2}+h\right]\cup [\pi -h,\pi +h]\right\}$ , and the initial default value of *h* is π/9.

The entire boundary reconstruction process is conducted on MATLAB. When fitting the curve, we use MATLAB’s curve fitting toolbox. The selected fitting method is smoothing splines, and the degree of curve fitting can be adjusted according to the smoothing parameter p (0≤p ≤ 1).

#### 2.2.4 Evaluation Method

In our experiment, the Dice similarity coefficient (DSC) and Hausdorff distance(HD) are used to evaluate the performance of the segmentation. DSC is a spatial overlap index, which can evaluate the similarity between the prediction area and the ground truth area. Its expressions are as follows.


(7) $$DSC=\frac{2*TP}{2*TP+FP+FN}$$

Where *TP* (true positive), *TN* (true negative), *FP* (false positive), and *FN* (false negative) can be calculated easily.

The Hausdorff distance is the maximum distance between two closed and bounded subsets. It is more sensitive to the segmented boundary. Its expressions are as follows.


(8) $$DH(X,Y)=\max\{d_{XY},d_{YX}\}=\max\left\{\underset{x\in X}{\max}\underset{y\in Y}{\min}d\{x,y\},\underset{y\in Y}{\max}\underset{x\in X}{\min}d\{x,y\}\right\}$$

where, *X* and *Y* are ground truth and segmented result, and *x*, *y* are points on *X* and *Y* respectively. *d{x,y}* is the Euclidean distance between *x* and *y*.

## 3 Results

**Table 3** shows the evaluation results of different data augmentation methods in Mask-RCNN and U-Net segmentation. In addition to the data augmentation method proposed in this paper, the traditional data augmentation methods (cropping, rotating, and mirroring) were used as the reference group. The specific experimental design is as follows:

− Baseline: Without any augmentation method, the training set is the original image data set;
− RefMeth_1: Cropping augmentation. In this experiment, all the tumor parts were included. The training set contains baseline data, and the amount of data is four times that of baseline;
− RefMeth_2: Rotating augmentation. In this experiment, the expanded rotation angles are 90°, 180°, and 270°. The training set contains baseline data, and the amount of data is four times that of baseline;
− RefMeth_3: Mirroring augmentation, including vertical, horizontal, and diagonal mirroring. The training set contains baseline data, and the amount of data is four times that of baseline;
− RefMeth_4: Combination of RefMeth_1, RefMeth_2, and RefMeth_3. The training set contains baseline data, and the amount of data is ten times of baseline;
− PropMeth_1: HVCMA. The training set only contains the augmented data, and the amount of data is four times of baseline;
− PropMeth_2: DCMA. The training set only contains the augmented data, and the amount of data is four times of baseline;
− PropMeth_3: Combination of PropMeth_1 and PropMeth_2. It can compensate for the information lost during the previous cutting. The amount of data is eight times of baseline.

**Table 3:** Segmentation performance evaluation on Mask-RCNN and U-Net.

Methods	Mask-RCNN		U-Net	
	DSC (%)	HD	DSC (%)	HD
Baseline	75.70 ± 3.792	121.75 ± 44.292	64.87 ± 2.066	159.48 ± 15.185
RefMeth_1	75.98 ± 3.271	110.84 ± 14.223	65.28 ± 2.245	179.32 ± 6.2712
RefMeth_2	77.34 ± 1.774	79.803 ± 8.744	65.55 ± 1.757	164.46 ± 7.9453
RefMeth_3	78.88 ± 3.000	73.920 ± 13.632	63.82 ± 3.299	169.90 ± 9.9893
RefMeth_4	80.52 ± 2.718	66.865 ± 14.626	66.85 ± 3.422	171.41 ± 11.353
PropMeth_1	85.36 ± 1.156	43.523 ± 2.7343	77.30 ± 2.900	66.869 ± 4.6270
PropMeth_2	85.16 ± 1.459	44.410 ± 6.5669	78.61 ± 2.616	59.627 ± 8.068
PropMeth_3	85.44 ± 1.062	44.641 ± 4.7393	79.08 ± 2.654	66.070 ± 2.1231

**Figure 8:**
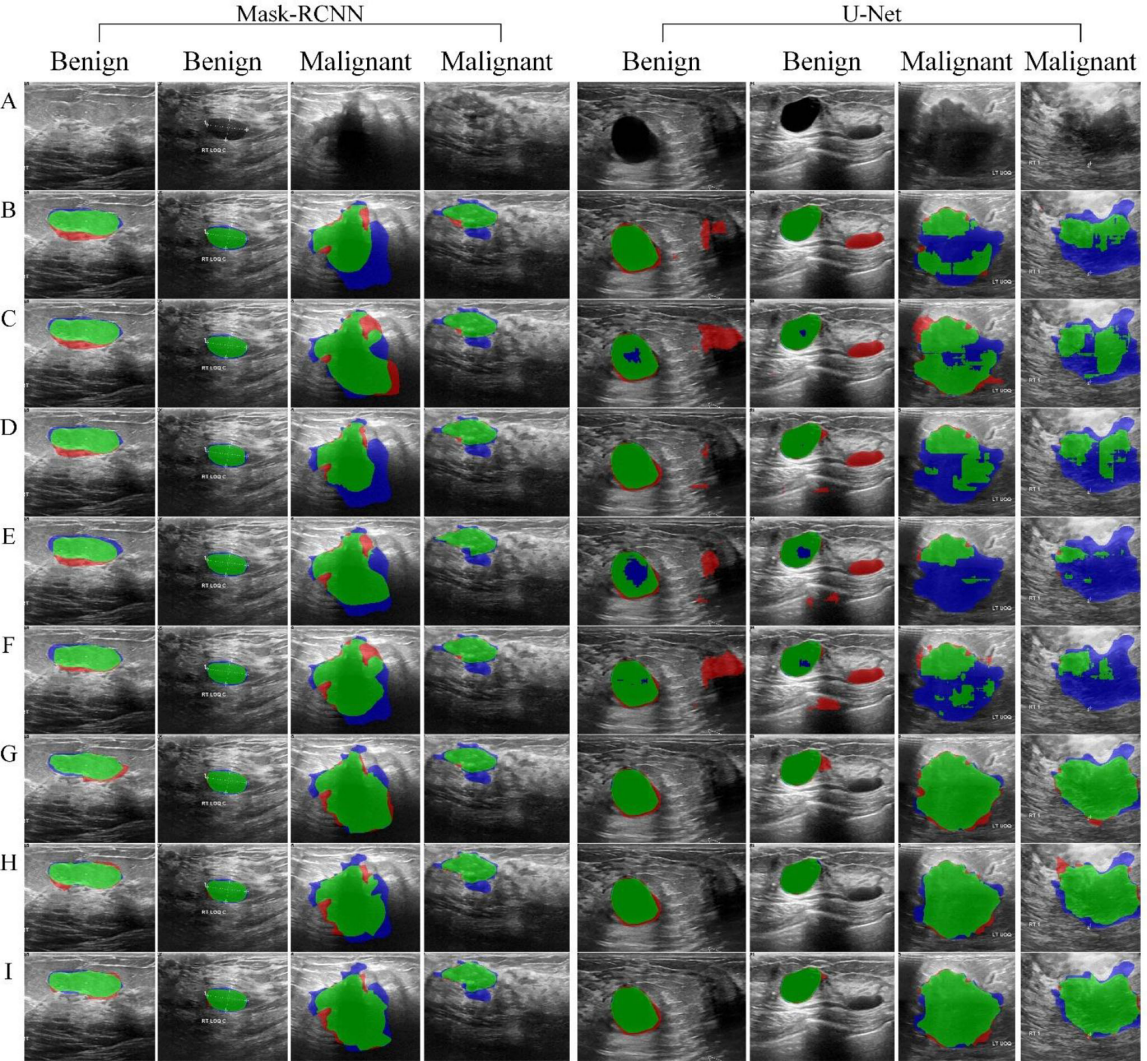
Tumor segmentation results. The blue area refers to the ground truth, the red refers to the prediction result, and the green refers to the overlap area. **(A)** original image, **(B)** Baseline, **(C)** RefMeth_1, **(D)** RefMeth_2, **(E)** RefMeth_3, **(F)** RefMeth_4, **(G)** PropMeth_1, **(H)** PropMeth _2, **(I)** PropMeth _3.

**Figure 8** shows the segmentation results based on Mask-RCNN and U-Net network, respectively, using different data augmentation methods.

## 4 Discussion

As shown in **Table 3**, PropMeth_1 and PropMeth_2 for single tumor segmentation proposed in this paper can improve the DSC value by 9.66% and 9.46% based on Mask-RCNN compared with the baseline result. However, the experimental group with the same amount of augmented data (RefMeth_1, RefMeth_2, and RefMeth_3) can only improve the DSC by 0.28%, 1.64%, and 3.18%, respectively. There is no noticeable improvement in the cropping augmentation method. As for the experimental group RefMeth_4, the DSC value can only increase by 4.82% with the data ten times of baseline.

With U-Net, PropMeth_1 and PropMeth_2 can increase the average DSC value by 12.43% and 13.74% compared with baseline results. The experimental group of RefMeth_1, RefMeth_2, RefMeth_3 and RefMeth_4 could only increase the average DSC value by 0.41%, 0.68%, -1.05%, and 1.98%, respectively. The four groups’ improvements are not as great as those based on Mask-RCNN, and even a negative effect occurred in RefMeth_3.

The experimental results show that RefMeth_1, RefMeth_2, and RefMeth_3 have litter improvement on U-Net. Moreover, RefMeth_4 is better than that of the single augmentation method. What is more, the improvement of the proposed method is about two times the combination of traditional methods, although the data volume is only 40%. Compared with U-Net, Mask-RCNN has similar results in different data augmentation groups. Therefore, the data augmentation method for single tumor segmentation proposed in this paper has strong applicability in Mask-RCNN and U-Net.

It can be seen from **Table 4** that the segmentation performance of the proposed method improved significantly in terms of benign and malignant tumors. In general, the segmentation performance of benign tumors is better than that of malignant ones. However, it is worth mentioning that the segmentation based on mask-RCNN shows that the DSC of malignant tumors is 16.36% higher than that of the baseline.

**Table 4:** Segmentation performance evaluation between benign and malignant tumors in DSC(%).

Methods	Mask-RCNN		U-Net	
	Benign	Malignant	Benign	Malignant
Baseline	80.33 ± 4.997	68.22 ± 8.047	65.66 ± 2.962	63.60 ± 3.767
RefMeth_1	78.81 ± 5.435	71.51 ± 3.566	66.16 ± 4.266	63.84 ± 6.313
RefMeth_2	80.76 ± 3.115	71.85 ± 5.060	67.56 ± 3.148	62.20 ± 4.988
RefMeth_3	81.49 ± 5.465	74.80 ± 3.377	66.74 ± 6.118	59.08 ± 1.969
RefMeth_4	84.00 ± 4.938	77.48 ± 7.164	68.23 ± 5.617	64.73 ± 4.333
PropMeth_1	88.07 ± 1.909	81.16 ± 1.681	79.18 ± 3.699	74.22 ± 4.886
PropMeth_2	87.64 ± 2.100	81.13 ± 2.234	79.52 ± 4.384	77.13 ± 2.542
PropMeth_3	87.89 ± 1.759	84.58 ± 1.510	79.93 ± 3.434	76.25 ± 4.010

In addition, a Wilcoxon signed-rank test was conducted to test the statistical significance of the DSC results of different methods. A p-value less than 0.05 is considered as statistical significance. The results are shown in **Table 5**.

**Table 5:** Results of statistical significance of DSC.

Group1	Group2	P-value on Mask-RCNN	P-value on U-Net
PropMeth_1	Baseline	0.000	0.000
PropMeth_1	RefMeth_1	0.000	0.000
PropMeth_1	RefMeth_2	0.000	0.000
PropMeth_1	RefMeth_3	0.001	0.000
PropMeth_1	RefMeth_4	0.180	0.000
PropMeth_1	PropMeth_2	0.003	0.007
PropMeth_1	PropMeth_3	0.621	0.000
PropMeth_2	Baseline	0.000	0.000
PropMeth_2	RefMeth_1	0.000	0.000
PropMeth_2	RefMeth_2	0.000	0.000
PropMeth_2	RefMeth_3	0.000	0.000
PropMeth_2	RefMeth_4	0.004	0.000
PropMeth_2	PropMeth_3	0.008	0.248
PropMeth_3	Baseline	0.000	0.000
PropMeth_3	RefMeth_1	0.001	0.000
PropMeth_3	RefMeth_2	0.000	0.000
PropMeth_3	RefMeth_3	0.005	0.000
PropMeth_3	RefMeth_4	0.068	0.000

**Figure 9:**
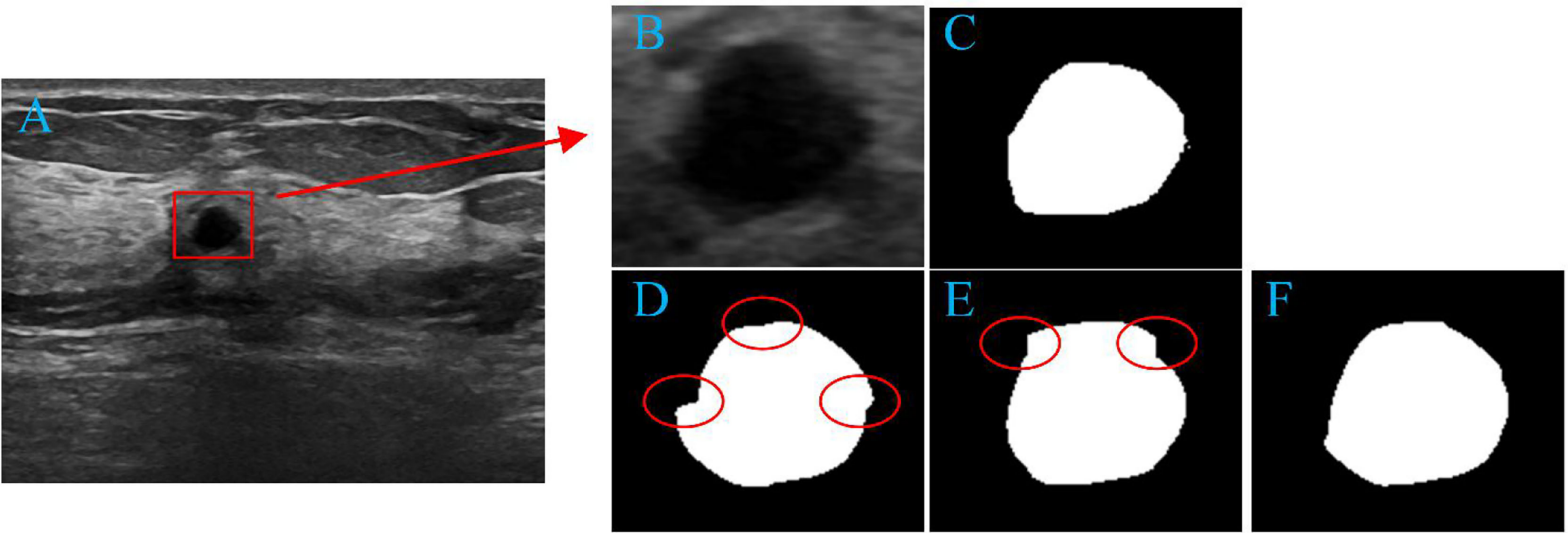
Tumor segmentation results. **(A)** original image, **(B)** tumor area, **(C)** the ground truth of tumor area, **(D)** the result of PropMeth_1, **(E)** the result of PropMeth_2, **(F)** the result of PropMeth_3.

PropMeth_3 is the result of boundary compensation and reconstruction. According to **Table 3**, the DSC value of PropMeth_3 is slightly increased compared with PropMeth_1 and PropMeth_2, as shown in **Figure 9**. The red circles in **Figures 9D, E** are the boundary discontinuity caused by the loss of information during cutting. The boundary is obtained ideally after boundary compensation and reconstruction, as shown in **Figure 9F**.

The proposed method for single tumor segmentation achieves better performance than traditional methods because the augmented data set has better sample independence. Traditional methods have the same pixel adjacent relationship in different samples, whether cropping, rotating, or mirroring. The proposed method in this paper does not have this problem for different image samples. Therefore, this method can obtain better tumor segmentation performance.

## 5 Conclusion

This paper studies the data augmentation method for single tumor segmentation. We applied this method to the public breast cancer ultrasound data set for tumor segmentation based on Mask-RCNN and U-Net. Experimental results show that the proposed method’s performance is much improved than the conventional data augmentation method. Furthermore, this method can also be applied to other single tumor segmentation or classification problems using other deep learning networks.

## Data Availability Statement

The original contributions presented in the study are included in the article/supplementary material. Further inquiries can be directed to the corresponding authors.

## Ethics Statement

The data set used in this article is a public data set, which has been provided ethical approval. The patients/participants provided their written informed consent to participate in this study.

## Author Contributions

LH and LJ proposed the idea of this study. ZC and BN conducted the experiments, carried out the data analysis, and wrote the manuscript. SH helped with the data analysis. LH and LJ revised the manuscript. QW and ZS participated in the discussion of research approach. All authors contributed to the article and approved the submitted version.

## Funding

Guizhou Province Science and Technology Project (grant Qiankehezhicheng (2019) 2794); Fundamental Research Funds for the Central Universities (N2119003); National Natural Science Foundation of China (NSFC) (grant 61701103); Science and Technology Fund Project of Guizhou Health Commission, No. (GZWKJ2021-377); Natural Science Foundation of Liaoning Province (grant 2019-ZD-0005).

## Conflict of Interest

The authors declare that the research was conducted in the absence of any commercial or financial relationships that could be construed as a potential conflict of interest.

## Publisher’s Note

All claims expressed in this article are solely those of the authors and do not necessarily represent those of their affiliated organizations, or those of the publisher, the editors and the reviewers. Any product that may be evaluated in this article, or claim that may be made by its manufacturer, is not guaranteed or endorsed by the publisher.
